# Sex-stratified osteochondral organ-on-chip model reveals sex-specific responses to inflammatory stimulation

**DOI:** 10.1016/j.mtbio.2025.101728

**Published:** 2025-04-02

**Authors:** Francisco Conceição, João Meneses, Filipa Lebre, Malin Becker, Nuno Araújo-Gomes, Rianne Vos, Ana R. Ribeiro, Ernesto Alfaro-Moreno, Jeroen Leijten, Liliana Moreira Teixeira

**Affiliations:** a Department of Bioengineering Technologies, Faculty of Science and Technology, TechMedCentre, University of Twente, 7522 NB, Enschede, the Netherlands; b Nanosafety Group, International Iberian Nanotechnology Laboratory, 4715-330, Braga, Portugal; c Optics11 Life, 1101 BM, Amsterdam, the Netherlands; d Organ-on-Chip Centre Twente, TechMed Centre, MESA+, University of Twente, 7522 NB, Enschede, the Netherlands

**Keywords:** Organ-on-chip, Osteoarthritis, Sex differences, Metabolic labeling, Chondrocytes, Inflammation, Extracellular matrix

## Abstract

Osteoarthritis (OA) is a musculoskeletal degenerative disease characterized by alterations in cartilage and subchondral bone leading to impaired joint function. OA disproportionally affects females more than males, yet the molecular mechanisms underlying these biological sex differences remain elusive. Current therapeutic strategies to halt the progression of OA are still lacking, in part due to the limited predictive potential of standard models which often do not account for sex disparities. Herein, an organ-on-chip microfluidic platform was developed to model the osteochondral unit, composed of adjacent bone and cartilage culture chambers, and capture sex-specific hallmarks of OA. Sex-stratified human primary chondrocytes and osteoblasts were compartmentalized within biomimetic hydrogels emulating the bone-cartilage interface, which were subjected to inflammatory triggers to mimic the onset of OA. We confirmed that interleukin-1β and Tumor Necrosis Factor-α stimulation triggered upregulation of pro-inflammatory cytokines and matrix metalloproteinases related genes in all donors, with marginal trends for increased expression in female cells. In addition, metabolic labeling coupled with confocal imaging revealed that inflammatory stimulation modulated extracellular matrix deposition by human chondrocytes in a sex-specific fashion. Not only matrix deposition but also matrix remodeling was altered upon inflammation, leading to a significant reduction in matrix stiffness in both cartilage and bone compartments. Overall, sex-stratified osteochondral unit on-chips offer novel insights into sex-specific cellular responses to inflammatory insults, demonstrating the importance of incorporating sex stratification in emergent organ-on-chip models. Thus, this platform provides a physiologically relevant 3D microenvironment to further investigate sex-specific drivers of OA, paving the way for targeted therapies.

## Highlights

• The molecular basis of sex-differences in osteoarthritis is not yet fully uncovered.
• A sex-stratified human organ-on-chip was used to mimic the osteochondral interface.
• Inflammation induces sex-specific differences in protein production and deposition.
• Nascent protein quantification and nanoindentation were adapted to organ-on-chip.

## Graphical abstract

**Figure:**
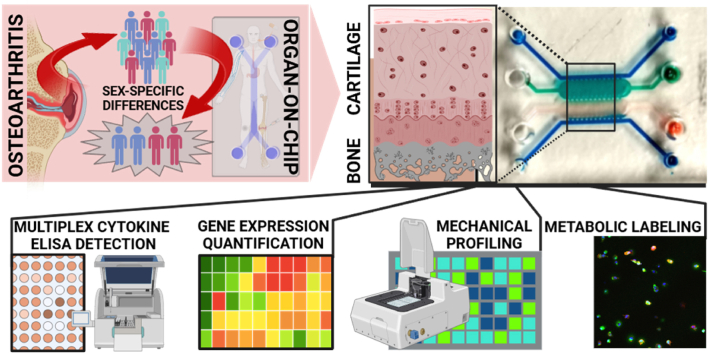

## 1 Introduction

Osteoarthritis (OA) is one of the most common musculoskeletal degenerative diseases that primarily affects load-bearing joints, leading to debilitating pain, swelling, and loss of joint function. Although OA can occur in almost all demographic groups, it is most prevalent in aged individuals and disproportionately affects females more than men [1]. OA pathophysiology is complex and heterogenous as it involves not only changes in cellular biology, but also alterations in joint tissue interactions such as synovium, menisci, tendons, ligaments, and subchondral bone, which potentiate OA onset and progression [2]. Multiple studies have reported that dysregulation of subchondral bone plays a key role in OA initiation and development [[3], [4], [5], [6], [7]]. Nonetheless, the molecular events underlying OA pathogenesis, and particularly the molecular basis of biological sex differences, have not yet been fully uncovered.

While animal models remain the gold standard when modeling OA, interspecies anatomical, genetic, and molecular disparities limit the successful translation of in vivo findings to clinical trials with human patients [8]. On the other hand, classical *in vitro* models are too often over-simplistic and do not replicate the native features of the joint microenvironment [9], which decreases their predictive potential for OA drug screening and disease modeling. To circumvent these limitations, organ-on-chip (OoC) models have emerged in the past decade as a promising alternative to conventional *in vitro* and in vivo models. OoC are microfluidic-based, three-dimensional (3D) humanized tools with the ability to faithfully replicate key features of articular joint function and microenvironment [10]. In addition, OoC provides precise control over complex variables, such as cellular and physico-chemical microenvironments. It also enables the integration of high-content analytical methods and advanced technologies for enhanced characterization and output generation [[11], [12], [13]]. While several OoC models have been reported for the modeling of cartilage during the onset of OA, only a few studies have addressed cell-cell communications between multiple joint tissue components [[14], [15], [16], [17]]. Notably, although the incidence and severity of OA is increased in female patients, sex-stratification is clearly underrepresented in existing OoC models.

In this study, we aimed to replicate the close interactions between osteoblasts and chondrocytes in the osteochondral compartment and demonstrate the applicability of OoC models to recapitulate sex-specific hallmarks of OA. The osteochondral unit on-chip model described here incorporates sex-stratified human primary cells within 3D microenvironments, providing a more accurate *in vitro* model. Taking advantage of the favorable optical access of the osteochondral unit-on-chip and its compatibility with standard molecular biology assays, we successfully recapitulated sex-specific cellular responses to inflammatory triggers, particularly in gene expression and secretion of pro-inflammatory markers, as well as in altered matrix deposition and remodeling. The herein proposed osteochondral unit on-chip model highlights the potential of organ-on-chip models to further investigate the sex-specific drivers of OA, ultimately leading to the development of sex-specific targeted therapies for OA.

## 2 Materials and methods

### 2.1 Cell culture

Human primary chondrocytes were isolated from cartilage from patients undergoing total knee replacement surgery. The collection and use of human cartilage surgical waste was approved by institutional ethics committee (Medisch Ethische Toetsingscommissie [METc] dossier number 2020–7255). Informed consent was obtained from all patients for the use of these tissues/cells. Human chondrocytes from 3 female (66–78 year old) and 3 male (62–81 year old) patients were isolated from knee articular cartilage. Chondrocytes were then expanded in chondrocyte expansion medium composed of Dulbecco's modified Eagle's medium (DMEM) high glucose (Gibco) supplemented with 100 U/mL penicillin/streptomycin (Pen/Strep, Gibco), 10 % Fetal Bovine Serum (FBS, Sigma-Aldrich), 1 mM sodium pyruvate (Gibco), 10 mM HEPES buffer (Gibco), 0.2 mM non-essential aminoacid mix (Gibco), 0.2 mM ascorbic acid (Sigma-Aldrich) and 0.4 mM proline (Sigma-Aldrich) at 37 °C and 5 % CO_2_ in an humidified incubator, changing medium twice a week until 80 % confluence. Chondrocytes up to passage 5 were used in this study.

Human primary osteoblasts were isolated from surgical waste from mandibular reconstruction surgery. The collection and use of human bone surgical waste was approved by institutional ethics committee (METc dossier number 2016.105). Informed consent was obtained from all patients for the use of these tissues/cells. In brief, fibula bone from female and male donors (23 and 63 year old, respectively) patients were cut into small pieces of approximately 20 mm^3^ and incubated with 2 mg/mL collagenase II (Worthington) solution for 1 h at 37 °C. Bone pieces were then washed vigorously with PBS and digested once more in collagenase II solution for 1 h at 37 °C. After another washing step with PBS, bone fragments were placed on a T75 flask and incubated in Minimum Essential Medium α (αMEM) supplemented with 10 % FBS and 100 U/mL Pen/Strep at 37 °C and 5 % CO_2_ in an humidified incubator, changing medium once a week until outgrowths of osteoblasts were visible in the flask. Osteoblasts up to passage 5 were used in this study.

### 2.2 Microfluidic chip fabrication

For device fabrication, master molds were micropatterned with SU-8 with a design adapted from other devices previously established in our department [18]. The device was composed of two cellular compartments measuring 5 mm long and 1.2 mm width each, interspaced by trapezoid pillars of 80 × 200 μm and flanked by perfusion channels 250 μm wide. Briefly, h100i orientation silicon wafers (Okmetic) were spin-coated with an SU-8100 negative photoresist (Microchem) at 500 rpm for 30 s and ramped up to 1500 rpm for 30 s, so that the average thickness of the SU-8 was of ∼250 μm. The SU-8 photoresist was then patterned by exposure to UV light with a 365 nm longpass filter using an EVG 6200NT mask aligner (EVGroup, Austria). The patterned wafers were then developed in RER600 (Fujifilm) followed by spraying, spinning, and drying. Finally, patterned wafers were washed with isopropanol (IPA) and dried using a stream of nitrogen gas.

Microfluidic chip devices were produced by soft lithography using polydimethylsiloxane (PDMS). Curing agent (Sylgard 184, Dow Corning) and PDMS prepolymer were mixed in a 1:10 ratio, degassed and poured onto SU-8 molds. PDMS was then cured at 65 °C in an oven for 2 h. The following day, the patterned PDMS was peeled from the SU-8 wafer and cut to the defined shape. The cell chamber inlets/outlets and the perfusion (media) inlets/outlets were punched with 1,5 and 1 mm biopsy punchers, respectively. Each PDMS chip was then oxygen plasma-bonded (Cute plasma oven, Femto Science, South Korea) to a glass/cover slide and stored at room temperature (RT) until further use. The day before use, chips were incubated at 65 °C overnight to revert to hydrophobic behavior. Prior to cell seeding/hydrogel injection, chips were sterilized under UV light for 15 min.

### 2.3 Hydrogel preparation and diffusion studies

Gelatin Methacryloyl (GelMA, Cellink) hydrogels were fabricated by mixing GelMA solution with the photoinitiator lithium phenyl-2,4,6-trimethylbenzoylphosphinate (LAP, Sigma-Aldrich) and dissolved in sterile PBS in a final concentration of 8 % (w/v) and 0.2 % (w/v), respectively. For the bone compartment, GelMA 8 % (w/v) was doped with 0.5 % nanohydroxyapatite (nanoHA, formulated as described elsewhere [19]). 5 μL of GelMA-nanoHA precursor solution was injected into the bone compartment and crosslinked under UV light for 90 s. 5 μL of GelMA precursor solution was then injected into the cartilage compartment and prior to crosslinking, the bone compartment was covered with aluminum foil to avoid excessive crosslinking. The chip was exposed to UV light for another 90 s followed by PBS injection in the perfusion channels. Permeability of the hydrogels was tested by injecting two fluorophores of distinct molecular weights (Fluorescein isothiocyanate-dextran - MW 10 kDa and Fluorescein isothiocyanate-bovine serum albumin - MW 66 kDa, Sigma-Aldrich) in both perfusion channels. The injection occurred sequentially by pipetting manually the fluorophores into the perfusion channels after hydrogel crosslinking in the central chamber. Imaging and diffusion quantification was performed using an EVOS FL microscope (Thermo Fisher Scientific, United States) every 15 min for a period of 2 h 30 min.

### 2.4 Cell tracking and cell viability assay

After reaching 80 % confluence, osteoblasts and chondrocytes were washed with PBS and detached using trypsin-EDTA solution (0.25 %/0.1 mM, Gibco) for 5 min at 37 °C. Cells were then collected, centrifuged at 300 g for 3 min, resuspended in expansion medium and counted. To confirm proper cell confinement in their respective compartments, an osteoblast suspension of 4 × 10^6^ cells/mL was prepared and incubated with 10 μM CellTracker™ Green CMFDA (Invitrogen) for 30 min at 37 °C. In parallel, a chondrocyte suspension of 4 × 10^6^ cells/mL was prepared and incubated with 10 μM CellTracker™ Blue CMAC (Invitrogen) for 30 min at 37 °C. After incubation with cell trackers, cells were centrifuged and suspended in GelMA-nanoHA or GelMA alone, followed by injection in the microfluidic chip (20 000 cells/chip), and crosslinked using the same protocol as in the diffusion studies. Following crosslinking, osteoblast differentiation medium composed of α-MEM supplemented with 100 U/mL Pen/Strep, 0.2 mM ascorbic acid, 10 mM beta-glycerophosphate (Sigma-Aldrich) and 0.1 μM dexamethasone (Sigma-Aldrich) was assigned to the perfusion channel adjacent to the bone compartment, while chondrocyte differentiation medium composed of DMEM supplemented with 100 U/mL Pen/Strep, 1 mM sodium pyruvate, insulin/transferrin/selenium mixture 1X (Gibco), 0.2 mM ascorbic acid, 0.1 μM dexamethasone and 10 ng/mL transforming growth factor beta 3 (TGF-β3, Peprotech) was assigned to the perfusion channel adjacent to the cartilage compartment. Cells were incubated overnight and then imaged using the EVOS FL microscope.

For viability assays, chondrocytes and osteoblasts were seeded in the microfluidic chip with the same protocol as in the cell tracker assay. Cells were cultured in osteoblast/chondrocyte differentiation medium for one week, refreshing medium every two days. To induce a pro-inflammatory environment, each differentiation medium was supplemented with 10 ng/mL interleukin 1β (IL-1β, Peprotech) and 10 ng/mL tumor necrosis factor α (TNF-α, Peprotech). After one week, cell viability was assessed by addition of 4 μM calcein-AM and 4 μM ethidium homodimer (Thermo Fisher Scientific) in PBS to the perfusion channels followed by incubation for 30 min at dark with 37 °C. Excess stain was washed with PBS and cells were imaged using an EVOS FL microscope. The percentage of viable cells was assessed by quantifying the percentage of Calcein-AM stained cells (green), divided by the total number of stained cells (Calcein-AM (green) + ethidium homodimer-1 (red)). For each condition, two pictures of each cell compartment (four pictures per chip) were taken and >250 cells per picture were considered for quantification. Cells were segmented and quantified with ImageJ software (v. 1.54f, NIH, USA).

### 2.5 Quantitative real-time polymerase chain reaction (qRT-PCR) analysis

Sex-matched chondrocytes and osteoblasts were seeded in GelMA or GelMA-nanoHA in the cartilage and bone compartment, respectively, at a density of 20 000 cells/chip. Osteoblast and chondrocyte differentiation medium was injected in the perfusion channel adjacent to the bone or cartilage compartment, respectively. Osteochondral units were cultured for 3 or 7 days under control conditions or under stimulation with 10 ng/mL IL-1β and TNF-α. For static cultures, the medium in each perfusion channel was refreshed every two days. For perfusion conditions, 3 mL syringes were pre-filled with differentiation medium and mounted on a SPLab syringe pump (Infusetek, China), and each syringe was then connected to each respective perfusion channel inlet. Perfusion setup was assembled and transferred to the incubator, after which perfusion was initiated at a rate of 25 μL/h.

At the end of each experiment, collagenase II solution (2 mg/mL) was injected in each perfusion channel and incubated for 30 min at 37 °C to degrade the GelMA hydrogels and release the cells. Cells from two chips for each condition were collected via aspiration and pooled with fresh differentiation medium. After centrifugation at 300*g* and supernatant removal, cells were lysed with lysis buffer (Machery-Nagel) and snap frozen at −80 °C. Total RNA from three independent experiments was extracted using the NucleoSpin RNA Mini kit, according to the manufacturer's protocol (Macherey-Nagel). RNA final concentration and purity (OD_260/280_) was determined using a NanoDrop 2000 instrument (NanoDrop Technologies). RNA was reverse transcribed into cDNA using the iScript cDNA Synthesis Kit (Bio-Rad), according to the manufacturer's protocol. qRT-PCR experiments were run using an iCycler iQ5 PCR thermal cycler (Bio-Rad Laboratories) and analyzed with the CFX Manager™ software (Bio-Rad). Target gene expression was quantified using the cycle threshold (Ct) values and relative mRNA expression levels were calculated as follows: 2^(Ct reference gene − Ct target gene). Human 18S ribosomal RNA was used as housekeeping gene. The primers used in the analysis are listed in Table S1.

### 2.6 Measurement of cytokines and chemokines by Luminex multiplex array

Conditioned medium from the perfusion channels was collected at day 3 and 7, from the osteoblast and chondrocyte channel independently, and snap frozen at −80 °C until analysis. The levels of cytokines/chemokines in undiluted conditioned medium from three independent female and male osteochondral units were measured by using a Procartaplex™ Human Inflammation 20-plex panel (Invitrogen), according to manufacturer's instructions. The panel consists of the following analytes: GM-CSF, IFN alpha, IFN gamma, IL-1 alpha, IL-1 beta, IL-4, IL-6, IL-8, IL-10, IL-12p70, IL-13, IL-17A, TNF alpha, IP-10 (CXCL10), MCP-1 (CCL2), MIP-1 alpha (CCL3), MIP-1 beta (CCL4), ICAM-1, sCD62E (E-selectin), sCD62P (P-selectin). Samples were measured with a Luminex™ FLEXMAP 3D™ (Diasorin) and data analysis was performed using Belysa® Immunoassay Curve Fitting Software (Merck Millipore).

An Artificial Intelligence (AI) based pipeline was used to uncover biological meaningful patterns in cytokine and chemokine data, enabling the understanding of (i) inter-sample variability and (ii) cytokines/chemokine correlation and relationship. The AI-based pipeline was developed using Python (v3.9.12) in a Jupyter Notebook environment (v6.5.4), using libraries such as NumPy (v1.23.5) and Pandas (v1.5.3) for data manipulation; Matplotlib (v3.6.3) and Seaborn (v0.12.2) for visualization; SciPy (v1.10.1) for hierarchical clustering and dendrogram generation; scikit-learn (v1.2.0) for feature scaling and principal component analysis (PCA). Aiming to have a robust and broad data analysis pipeline, two datasets derived from FLEXMAP 3D™ system measurements were used, namely median fluorescence intensity (MFI) raw values and the converted analyte concentrations using Luminex's standard curves. Each original dataset contains 48 observations (N = 48) on 20 protein targets, with measurements spanning a wide range of inflammatory and immune-related cytokines and chemokines for time points 3 and 7. Both datasets include experimental labels, fostering a comprehensive clustering analysis of both samples and cytokines/chemokines. Due to a technical issue, one of the samples collected from chondrocyte perfusion channel in control conditions at day 7 had to be removed from the analysis. The MFI-based dataset was used to identify clustering patterns among the samples. Dimensionality reduction was performed using PCA, focusing on the first and second principal components (PC1 and PC2) to envision sample distribution and variability. The cytokines and chemokines contributing most significantly to PC1 and PC2 were identified, revealing major drivers of inter-sample differences.

The derived concentration based dataset uncovered cytokines and chemokines with similar expression patterns. Data pre-process included (i) missing value imputation (i.e., missing values were replaced with 10 % of the minimum non-missing value for the corresponding analyte), (ii) removing null and low variance cytokines/chemokines (i.e., analytes with a variance below 0.9 were excluded), and data normalization (i.e., Z-score standardization was applied using StandardScaler function from the sci-kit-learn library [software version 1.2.0]), scaling each analyte to zero mean and unit variance for comparability. Afterward, hierarchical clustering was performed on the normalized data using a correlation based distance metric and the complete linkage method. Lastly, a cluster map (i.e., a dendrogram integrating the Pairwise Pearson's correlations heatmap) was generated.

### 2.7 Nascent protein labeling and immunocytochemistry

To detect newly deposited proteins, also defined as nascent protein production, osteochondral units were cultured in nascent protein medium composed of glutamine-, methionine- and cystine-free high glucose DMEM supplemented with 1 X Glutamax (Gibco), 0.2 mM cystine (Sigma-Aldrich), 0.1 mM 4-Azido-L-Homoalanine (AHA, Jena Bioscience) [20]. Depending on the cell compartment, nascent protein medium was supplemented with osteoblast or chondrocyte differentiation supplements as in the cell viability experiments. For inflammatory conditions, 10 ng/mL IL-1β and TNF-α was added to the medium. After one week of culture under static or perfusion conditions, osteochondral units were washed twice in PBS with 0.1 % bovine serum albumin (BSA) and incubated with 30 μM DBCO-488 solution in 0.1 %BSA at 37 °C for 30 min and 5 % CO_2_. Chips were washed twice and fixed with 4 % paraformaldehyde at RT for 1h, followed by another two washes with 0.1 %BSA in PBS. Cells were permeabilized with 0.1 % Triton X-100 (Sigma-Aldrich) at RT for 15 min and then blocked with 0.1 %BSA for 1 h 30 min. Primary antibodies were diluted in 0.1 %BSA in PBS and incubated at 4 °C overnight. Antibodies and dilutions included anti-collagen type I (1:100, Novus Bio, NB600-450) and anti-collagen type II (1:100, Santa Cruz Biotechnology, sc-52658). After incubation, osteochondral units were washed thrice with 0.1 %BSA in PBS and incubated with a secondary antibody Alexa-Fluor 555 (Invitrogen, A32773) overnight at 4 °C. For F-actin staining, Phalloidin-594 (1:100, Biolegend, 424203) was diluted in 0.1 %BSA in PBS and incubated at 4 °C overnight. Lastly, after washing with 0.1 %BSA in PBS thrice, nuclei were counterstained with DAPI (1:1000, Biolegend, 422801) at RT for 15 min. Samples were kept in PBS at 4 °C in the dark until image acquisition.

### 2.8 Image acquisition and analysis

A Zeiss LSM880 confocal microscope was used to acquire z-stack images at 20X 0.8NA (0.42 μm per pixel) and 2 μm step size. 3D suite plugin [21] from ImageJ was used to segment and quantify nascent protein, actin and collagen I/II volume and signal intensity. Briefly, ∼100 μm z-stacks composite images were split into individual channels, and nuclei were segmented and used as seeds for the segmentation of nascent protein and actin of each cell. For quantification of the secreted nascent protein volume, each single cell was cropped, and the actin mask was subtracted from the nascent protein mask, generating a mask encompassing the extracellular matrix (ECM) only. If the subtraction resulted in an empty image, cells were considered to have nascent protein expression restricted to the intracellular region. The thresholding for segmentation was chosen by comparing different thresholds to the manual segmentation of three different stacks (Fig. S1). The percentage of collagen I/II positive cells was calculated by dividing the number of collagen positive cells by the total number of nuclei segmented on the image.

### 2.9 Nanoindentation measurements

For localized mechanical analysis, the chips were carefully delaminated, leaving the hydrogel intact in either the PDMS chamber or on the glass slide. Optical interferometry-based nanoindentation measurements were performed with a Pavone nanoindenter (Optics11 Life) at 37 °C in PBS. The gels were probed with a tip with a cantilever stiffness of ∼0.5 N/m and a tip radius of ∼25 μm. For spatial analysis, samples were imaged, and regions of interest were indicated for the measurements in the software. Then, matrix scans with a max load of 0.5 N/m applied at 10 μm/s or 30 μm/s were performed. For analysis, the Hertz Model was applied to fit the load indentation curve between 1 μm and 4 μm of indentation.

### 2.10 Statistical analysis

GraphPad Prism v10.3.1 was used for all statistical analysis. Unless otherwise stated in the figure legend, two-way ANOVA followed by Tukey's’s multiple comparison test was used to assess statistical significance, where differences between groups were considered significant when ∗p < 0.05, ∗∗p < 0.01, ∗∗∗p < 0.001, ∗∗∗∗p < 0.0001. All experiments were repeated as described in the figure legends. For image analysis, at least 4 images representative of different locations in the chip were used in the analysis for each independent experiment.

## 3 Results

### 3.1 Characterization of the osteochondral unit-on-chip

**Fig. 1:**
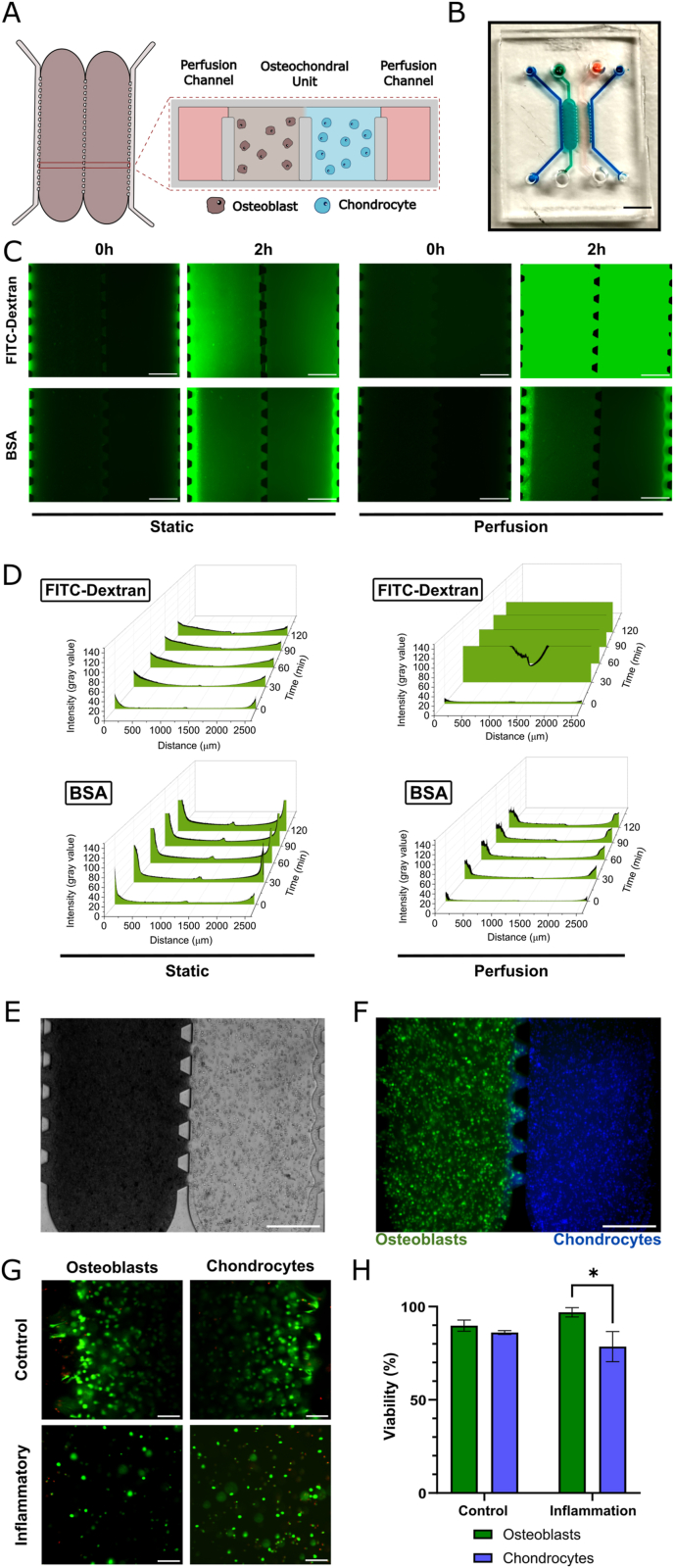
Design and characterization of the osteochondral unit-on-chip. A) Schematic representation of the chip, composed of two cellular chambers for osteoblasts and chondrocytes flanked by an array of pillars and two perfusion channels. B) Top view of the PDMS chip. Scale bar – 2 mm. C) Quantification of the diffusion of fluorescent molecules through GelMA hydrogels. After 2 h, FITC-Dextran 10 kDa (Top row) or BSA 63 kDa (Bottom row) diffuse through the entire hydrogel both under static and perfusion conditions (scale bar – 500 μm). D) Pixel intensity at longitudinal cross sections was plotted at different time points, over a total period of 150 min (Top row – FITC-Dextran 10 kDa; Bottom row – BSA - 66 kDa). E) Brightfield micrograph depicting the osteochondral unit-on-chip after seeding. Scale bar – 500 μm. F) The use of fluorescent cell trackers showed that both osteoblasts (green) and chondrocytes (blue) were confined to their respective compartments. Scale bar – 500 μm. G) Cellular viability was confirmed after 7 days of culture in both bone and cartilage compartments. Live cells – green; Dead cells – red. Scale bar – 100 μm. H) Viability was quantified after 7 days of culture, under control conditions or under inflammatory stimulus. Data from three osteochondral units was expressed as mean ± SD (Two-way ANOVA followed by Tukey's multiple comparisons test, ∗p < 0.05). (For interpretation of the references to color in this figure legend, the reader is referred to the Web version of this article.)

The osteochondral unit-on-chip was developed to recapitulate the interactions between osteoblasts and chondrocytes within a physiologically relevant 3D microenvironment. The device consists of two cellular compartments, separated by an array of micropillars and flanked by perfusion channels (Fig. 1A and B). The micropillar array between the compartments enabled unperturbed diffusion of secreted factors between both cell types, in close proximity. The dimensions of the cellular chambers are provided in Fig. S2.

Medium supply and metabolite exchange is a concern when using minute medium volumes in organ-on-chip models. To assess nutrient distribution within the hydrogel, we compared the diffusion kinetics of two fluorescent molecules with distinct molecular weights in both static incubation and dynamic perfusion conditions (Fig. 1C and D). Both FITC-Dextran (10 kDa) and FITC-BSA (66 kDa) fully diffused across the cellular compartments, with fluorescence detected at the center of the device within the timeframe of our experiment (120 min). Diffusion of FITC-Dextran under dynamic perfusion is markedly faster than in static incubation, as the fluorescence was completely saturated at the cellular compartments after 30 min.

Pillar arrays effectively constrained each cell-laden hydrogel to their respective compartments during seeding, while allowing for seamless cellular communication between the adjacent compartments, similar to their native counterparts. Given the differing mechanical properties of bone and cartilage in vivo, GelMA hydrogels in the bone compartment were supplemented with nanohydroxyapatite (nanoHA) to mimic the mineralized bone matrix. This modification resulted in a significant increase in matrix stiffness in the bone compartment relative to the cartilage compartment (Fig. S3).

After confirming hydrogel permeability, primary human osteoblasts were encapsulated in the nanoHA doped GelMA within the bone compartment, while primary human chondrocytes were seeded in GelMA within the adjacent cartilage compartment (Fig. 1E). Both cell types remained confined to their respective compartments throughout the experiments (Fig. 1F). After one week of culture, viability assays indicated that both osteoblasts and chondrocytes remained viable, even when exposed to pro-inflammatory conditions (Fig. 1G and H).

### 3.2 Female osteochondral units are more responsive to inflammatory triggers

**Fig. 2:**
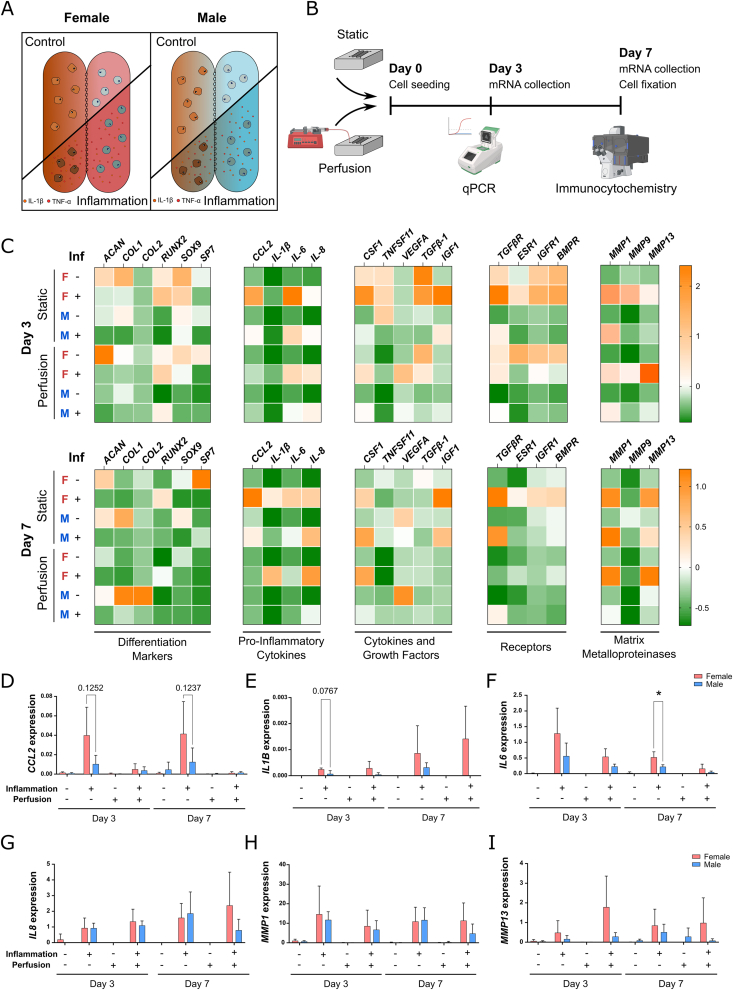
Inflammation promotes differential gene expression in female and male cells. A) Schematic representation of the experimental conditions. B) Experimental timeline. Osteochondral unit-on-chip devices seeded with female or male osteoblast/chondrocyte pairings were cultured for 7 days in static conditions or under perfusion. mRNA was collected at Day 3 and Day 7, and cells were fixed at Day 7 for immunocytochemistry staining. Icons from Biorender.com were used in figure. C) Heatmap depiction of gene expression of several differentiation markers, cytokines, growth factors, receptors and matrix remodeling enzymes. Data from three independent experiments is expressed as z-score and color coded as shown in the legend. D) Relative *CCL2*, E) *IL1B*, F) *IL6*, G) *IL8*, H) *MMP1* and I) *MMP13* gene expression of female and male osteoblast/chondrocyte pairings exposed to control or inflammatory stimulus, cultured either in static or perfusion conditions. Data from three independent experiments was expressed as mean ± SD (Two-way ANOVA followed by Tukey's multiple comparisons test, ∗p < 0.05). (For interpretation of the references to color in this figure legend, the reader is referred to the Web version of this article.)

To demonstrate the potential of the osteochondral unit on-chip model for sex-stratified disease modelling, primary chondrocytes and osteoblasts from both male and female human donors were seeded into their respective compartments. Osteoblast/chondrocyte pairs were cultured for one week under either control conditions or exposed to inflammatory stimuli to induce an OA-like microenvironment (Fig. 2A). Week one was chosen as the timepoint since pre-committed primary cells were used in our model, and differences in differentiation marker gene expression are apparent in the first week of culture (Fig. 2B, Fig. S4). It is well established that sex-specific differences in gene expression play a pivotal role in the differential progression of OA between males and females, both in vivo and in the clinical setting [[22], [23], [24]]. Taking this into account, we proceeded with gene expression analysis. mRNA was harvested at two timepoints—day 3 and day 7—to capture acute responses to inflammatory stimulation and evaluate its impact on gene expression. After the experiment, cells were fixed and processed for immunocytochemistry (Fig. 2B).

The expression profiles of several key genes, including chondrocyte/osteoblast differentiation markers, pro-inflammatory cytokines, growth factors, and matrix metalloproteinases (MMPs), are summarized in Fig. 2C. Notably, changes in differentiation markers and growth factor gene expression such as *ACAN* and *IGF-1* were more pronounced at day 3 under static conditions, indicating a rapid cellular response to the inflammatory stimulus (Fig. 2C, Fig. S5). Furthermore, treatment with IL-1β and TNF-α resulted in a general upregulation of pro-inflammatory cytokines and MMPs across all conditions (Fig. 2D–I).

Previous preclinical studies have reported elevated levels of cytokines, such as *CCL2*, *IL1B*, *IL6*, and *IL8*, in the serum or synovial fluid of female OA patients compared to males [25,26], highlighting the heightened inflammatory response in female donors. Consistent with these findings, we observed a trend towards increased expression of *CCL2*, *IL1B*, and *IL6* in female cells relative to male cells within our model, particularly under static culture conditions (Fig. 2D–F). Interestingly, *MMP1* and *MMP13* expression did not differ significantly between male and female cells under any condition (Fig. 2H and I), suggesting that MMP gene expression may not exhibit sex-dependent variation within the timeframe of our experiments.

### 3.3 Inflammation leads to augmented cytokine secretion in osteochondral units

**Fig. 3:**
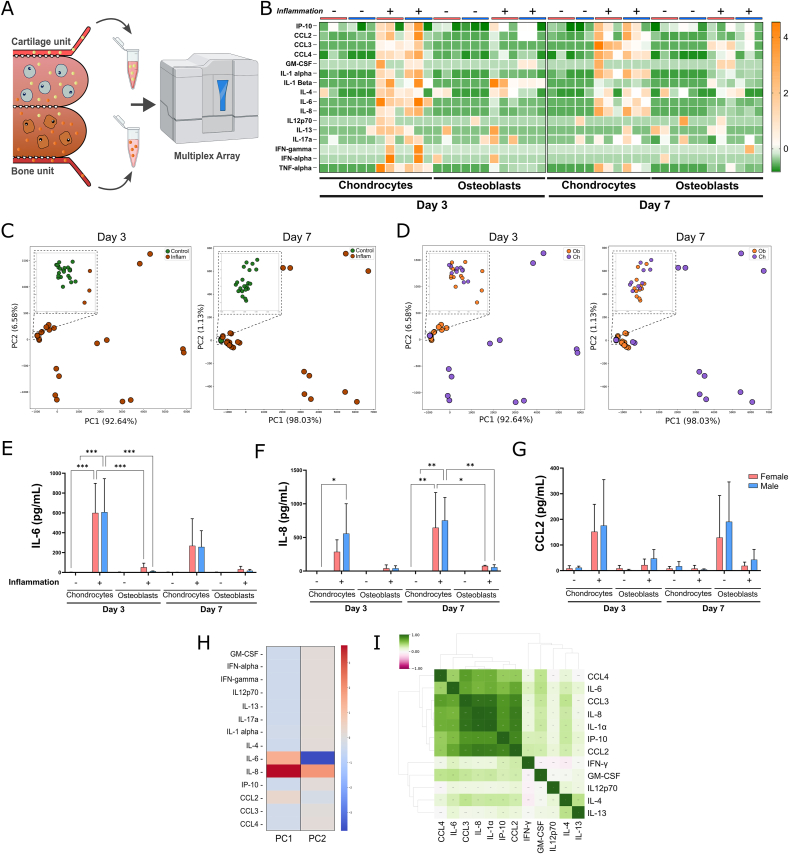
Inflammation induction leads to increased secretion of pro-inflammatory mediators. A) Perfused conditioned medium is collected independently from the chondrocyte and osteoblast outlet and screened using a multiplex cytokine/chemokine array. Icons from Biorender.com were used in figure. B) Heatmap depiction of inflammatory mediator levels in samples collected from chondrocyte and osteoblast outlets in two time points. Data from three independent experiments is expressed as z-score and color coded as shown in the legend. Light red bar – Female. Blue bar – male. C) PCA plot of all samples collected on day 3 (left) and day 7 (right) and stratified by culture condition. Inset represents a zoomed out area containing most of the baseline samples. D) PCA plot of all samples collected on day 3 (left) and day 7 (right) and stratified by cellular compartment outlet. Inset represents a zoomed out area containing most of the baseline samples. Ob-Osteoblast. Ch-Chondrocyte. E) IL-6, F) IL-8 and G) CCL2 concentration on samples collected from female and male osteoblast or chondrocyte exposed to baseline conditions or inflammatory stimulus. Data from three independent experiments was expressed as mean ± SD (Two-way ANOVA followed by Tukey's multiple comparisons test, ∗p < 0.05). H) Visualization of the contribution of specific inflammatory mediators to the variance of principal components 1 and 2 (PC1-2). I) Cluster map depicting the hierarchical correlation of the different inflammatory mediators. The map is color coded as shown in the legend. (For interpretation of the references to color in this figure legend, the reader is referred to the Web version of this article.)

Gene expression analysis showed trends towards an increase in the expression of pro-inflammatory genes in osteochondral units exposed to inflammatory triggers. To confirm that the observed gene expression effectively translates into altered secretion of pro-inflammatory mediators, we conducted a multiplex array on conditioned medium collected from osteochondral units (Fig. 3A). The levels of pro-inflammatory mediators are summarized in Fig. 3B. Inflammatory triggers stimulate the secretion of cytokines such as IL-6 and IL-8, consistent with the gene expression assays. Next, to investigate if the secretory profile can be used to differentiate the different conditions, the cytokine/chemokine measurements were analyzed by PCA. Of note, the levels of IL-1β and TNF-α were removed from the analysis since these are added to induce inflammation and might add unwanted bias when clustering the samples. The first two components of the PCA (which explain 94.4 % of the total variance) clearly separate samples from osteochondral units in baseline conditions and the ones exposed to inflammatory stimulus. (Fig. 3C).

As the perfusion channels flanking the osteoblast and chondrocyte compartments are individually addressable, similarly to previous established models [27], we then inquired whether we can differentiate between medium samples collected from either osteoblast or chondrocyte channels. While no specific clusters were identified in baseline conditions, medium samples from inflamed chondrocyte and osteoblast perfusion channels clustered separately with small overlap (Fig. 3D). This observation suggests that the secretory profile of the osteochondral units could be used to stratify the response from osteoblasts and chondrocytes to inflammatory triggers. We also investigated whether the variance explained by principal components is driven by one or more mediators. We found that the variance of principal component 1 and 2 is mostly driven by three different mediators (IL-6, IL-8 and CCL2) (Fig. 3H). The concentration of IL-6 and IL-8 is significantly increased in inflamed osteochondral units (Fig. 3E and F), while differences in CCL2 secretion did not reach statistical significance (Fig. 3G). Although significant differences between the levels of IL-6 and IL-8 in media collected from osteoblast and chondrocyte outlets, no significant differences were observed between male and female osteochondral units. In addition, no sex-specific clusters were identified in the PCA analysis (Fig. S6). While we observed a high variability between the different donors, which could explain the lack of significant sex-specific differences, induction of inflammation in the osteochondral units led to a robust increase in expression of pro-inflammatory mediators involved in immune recruitment and tissue remodeling [28,29]. When plotting the measurements of the multiplex array in a cluster map, we observed that the secretion profiles of cytokines/chemokines IL-6, IL-8, CCL2, CCL3 and CCL4 are highly correlated between them (Fig. 3I). These results highlight the ability of our model to recapitulate the pro-inflammatory microenvironment characteristic of the joint tissue during OA [28,30].

### 3.4 Sex-specific modulation of extracellular nascent protein deposition after induction of inflammation

**Fig. 4:**
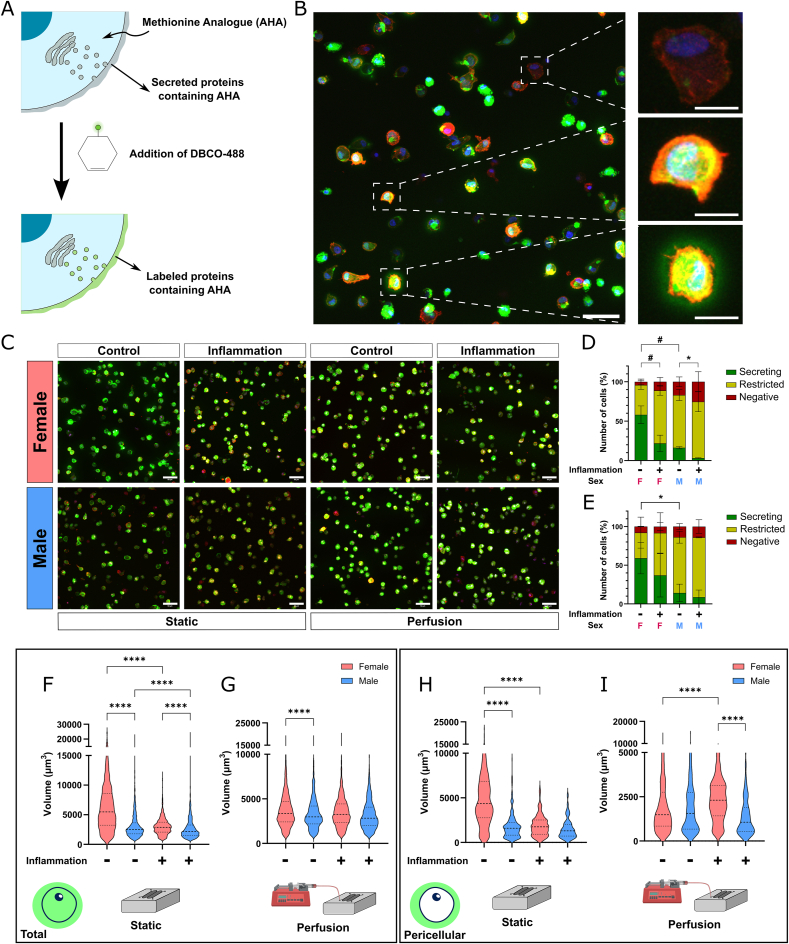
Sex-specific differences in chondrocyte protein secretion and deposition are apparent after exposure to inflammatory stimuli. A) Schematic representation of nascent protein labeling by Click-it chemistry. The methionine analog AHA in the culture media is incorporated into methionine-containing nascent proteins. In the end of the culture period, the addition of DBCO-modified fluorophore allows for visualization of nascent proteins. B) Micrograph of nascent protein staining in chondrocytes cultured for 7 days. Nuclei – Blue; Nascent protein – Green; Actin – Red. Scale bar – 50 μm. Insets represent negative cells (top), cells with nascent proteins restricted to the inside of the cell (middle) and cells that show protein deposition outside the cell (bottom). Scale bar – 10 μm. C) Representative overview of nascent protein deposition in female and male chondrocytes, after 7 days of culture under control or inflammatory conditions, under static or perfusion conditions. Nuclei – Blue; Nascent protein – Green; Actin – Red. Scale bar – 50 μm. D) Distribution of nascent protein deposition in female and male chondrocytes cultured under static or E) perfusion conditions. Data from up to 3 independent experiments is expressed as mean ± SD (Two-way ANOVA followed by Tukey's multiple comparison test, ∗p < 0.05 nascent protein outside, #p < 0.05 nascent protein inside). F) Total nascent protein volume in female and male chondrocytes cultured under static or G) perfusion conditions. Data from more than 1000 single cell data points pooled from 3 independent experiments is expressed in violin plots (Kruskal-Wallis followed by Dunn's multiple comparison test, ∗∗∗∗p < 0.0001). H) Quantification of the volume of nascent protein deposition outside the cells, in female and male chondrocytes cultured under static or I) perfusion conditions. Data from more than 1000 single cell data points pooled from 3 independent experiments is expressed in violin plots (Kruskal-Wallis followed by Dunn's multiple comparison test, ∗∗∗∗p < 0.0001). (For interpretation of the references to color in this figure legend, the reader is referred to the Web version of this article.)

Chondrocytes play a crucial role in maintaining the quality and function of cartilage ECM, a process often disrupted in OA. Thus, assessing protein deposition dynamics during cartilage remodeling directly on-chip is therefore essential for our model. Here, we employed a previously established metabolic labeling technique to visualize de novo protein deposition by chondrocytes, at the single cell level and with high spatial resolution [20]. Briefly, methionine analogs containing azide groups, AHA, added to the cell culture media, are incorporated into newly synthesized proteins (Fig. 4A). By binding a fluorophore-conjugated cyclooctyne (DBCO-488) to the azide groups via Click chemistry, we were able to visualize and quantify nascent methionine-containing proteins, including collagens and laminins.

After seven days of culture, DBCO labeling revealed nascent protein deposition by chondrocytes throughout the entire cartilage compartment (Fig. 4B). At the single cell level, protein deposition was notably heterogeneous. Within the same field of view, we observed chondrocytes with no detectable protein production, those with protein production limited to the intracellular region, and others depositing protein into the pericellular matrix (Fig. 4B). These cells will be hereafter referred to as negative, restricted, or secreting chondrocytes, respectively. Following this observation, to assess how inflammation affects nascent protein deposition dynamics, chondrocytes from both male and female donors were cultured under either control conditions or inflammatory stimuli (Fig. 4C). Nascent protein labeling and qualitative analysis showed that, under static conditions, inflammation induced a shift towards an increased proportion of restricted chondrocytes, reducing the ratio of secreting chondrocytes (Fig. 4D). Although this shift occurred in both male and female chondrocytes, female cells demonstrated a greater capacity for pericellular matrix deposition under baseline conditions. In contrast, no significant changes in the ratio of secreting chondrocytes were observed when osteochondral units were cultured under perfusion, though the baseline differences between male and female cells persisted (Fig. 4E).

As the straightforward optical access of the osteochondral unit allows for optimal imaging conditions, valuable information on protein deposition can be extracted and quantified at a single cell level in the 3D space (Fig. S7). After observing qualitative sex-specific differences in nascent protein secretion, we sought to determine whether total nascent protein volume also varied between sexes. To this end, we quantitatively analyzed nascent protein deposition in 3D in over 1000 single cells per condition. Under static conditions, inflammatory stimuli significantly reduced total nascent protein volume in both male and female chondrocytes (Fig. 4F). Notably, female chondrocytes exhibited significantly higher nascent protein production than male chondrocytes under both baseline and inflammatory conditions. However, when cultured under perfusion, inflammation did not induce significant changes in total nascent protein deposition (Fig. 4G). Interestingly, baseline sex-specific differences in nascent protein volume persisted, indicating an intrinsic higher protein production capacity in female chondrocytes that is independent of culture conditions.

While total nascent protein volume reflects the overall protein production capacity of each chondrocyte, subtle differences in pericellular protein secretion may be overlooked. Prior work using mesenchymal stromal cells has shown that cellular outcomes are influenced by both the initial engineered interface and the cell's adhesion to and remodeling of newly deposited local proteins shortly after cultures begin [20,31]. Yet, to the best of our knowledge, no similar study has been conducted using chondrocytes in close proximity with osteoblasts, in a physiologically relevant context, such as in an organ-on-chip platform. Given the critical role of matrix deposition in maintaining cartilage health, we analyzed the effect of inflammation on the volume of pericellular nascent protein deposition by ECM-secreting chondrocytes. After subtracting the intracellular component of nascent protein production, we found that female chondrocytes exhibited higher pericellular protein secretion compared to male cells at baseline under static conditions (Fig. 4H). Strikingly, inflammation significantly reduced pericellular protein secretion in female chondrocytes, while male chondrocytes showed no significant change. Conversely, under perfusion, female chondrocytes displayed an increased volume of pericellular protein secretion following exposure to inflammatory stimuli, underscoring the importance of fluid dynamics in modulating cellular behavior (Fig. 4I). In contrast, no statistically significant changes in male chondrocyte protein secretion were observed, further highlighting the sex-specific differences in sensitivity to inflammation. Given that during OA progression cartilage matrix composition changes as chondrocytes have been shown to shift from producing collagen II to collagen I^32^, we hypothesize that these differences might be driven by alterations in collagen production. Chondrocytes showed sex-specific differences in collagen I and II deposition, where inflammation leads to increased collagen I production in male chondrocytes while massively impacting the number of collagen II producing female chondrocytes (Fig. S8). These findings are in line with published transcriptome datasets of knee articular cartilage (OA and healthy samples), identifying a significant increase of matrix related proteins in response to OA in female cartilage compared to male cartilage [33].

### 3.5 Inflammation leads to a decline in ECM stiffness

**Fig. 5:**
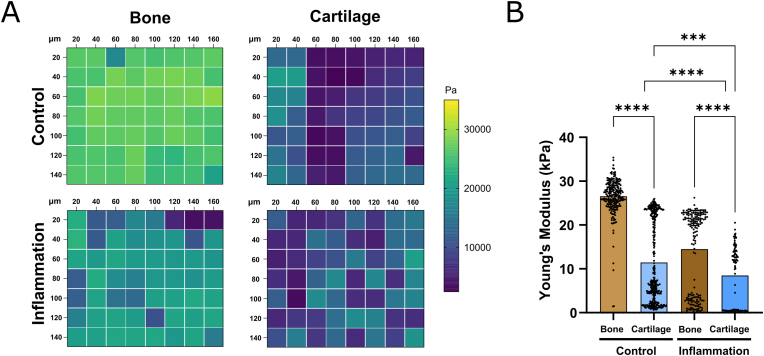
Inflammation leads to decreased hydrogel mechanical properties in both bone and cartilage compartments. A) Representative heatmaps depicting Young's Modulus array measurements from female osteochondral units, measured after 7 days of culture under perfusion. B) Young's Modulus from female osteochondral units cultured under perfusion. Data is expressed as mean of individual data points pooled from 4 different arrays (Kruskal-Wallis followed by Dunn's multiple comparison test, ∗∗∗p < 0.001, ∗∗∗∗p < 0.0001) from one independent experiment.

During the progression of OA, chondrocytes actively contribute to catabolic processes that ultimately lead to the degradation of the cartilage ECM [32]. This aligns with the changes in MMP gene expression we observed following exposure to inflammatory stimuli, prompting the hypothesis that increased MMP expression would result in ECM destabilization within the osteochondral units. To evaluate this, analytical measurement of the mechanical properties of the hydrogel with minimal sample processing is critical for assessing differential modulation of matrix remodeling. To assess this, we used high precision nanoindentation mapping to measure the ECM's mechanical properties. With this approach, we measured the Young's Modulus directly from the on-chip tissues from each of the compartments, cartilage and bone, both in control and inflamed conditions. Consistent with observations in empty hydrogels, the nanoHA-doped GelMA in the bone compartment exhibited significantly higher stiffness than GelMA alone in the cartilage compartment, both at baseline and following inflammation (Fig. 5A and B). This result highlights the presence of a clear stiffness boundary between the two compartments, maintaining the distinct mechanical properties of the bone and cartilage matrices even under inflammatory conditions. Furthermore, array measurements on the cell laden hydrogel surface indicated a decrease in matrix stiffness after inflammatory stimulation in both the bone and cartilage compartments (Fig. 5A and B), suggesting increased matrix fragility in both regions.

## 4 Discussion

OA is one of the most prevalent chronic musculoskeletal conditions in the elderly population, representing a considerable socioeconomic burden [34]. Of note, OA is more prevalent in females than in males, although the underlying causes of this disparity are still being investigated [[35], [36], [37], [38]]. To develop improved, sex-stratified therapeutic approaches for the management of OA, dissecting the complex molecular signaling pathways involved in OA pathophysiology is an important breakthrough.

While the joint is comprised of a multitude of different tissues, here we focused on modeling the interactions taking place at the osteochondral compartment. Existing OoC platforms that model the osteochondral tissue rely on gradients of differentiation factors to drive mesenchymal stromal cell differentiation and generating an osteogenic-chondrogenic interface [27,39,40]. Lin et al. demonstrated clear interactions between chondral and osseous components in response to IL-1β, resulting in robust MMP secretion and decreased anabolic gene expression [27]. Similar findings were observed when osteochondral tissues were generated after differentiation of induced pluripotent stem cells (iPSCs) [40].

However, although this strategy generates clear biphasic tissue differentiation, cell monitoring and compatibility with advanced microscopy tools is challenging due to difficult optical access to tissue chambers.

In the present study, contiguous cell chambers seeded with cell laden hydrogels were directly mounted on glass coverslips suited for confocal microscopy, allowing for quantification of protein deposition dynamics at single cell resolution while also being compatible with standard molecular biology assays. Using a combination of qRT-PCR, multiplex array protein quantification, metabolic labeling and nanoindentation, we sought to evaluate whether we can detect key sex-specific features of the early onset of OA. In our model, inflammatory stimulus led to a rapid decrease in aggrecan expression concomitant with an exacerbated expression of pro-inflammatory cytokines and MMPs. This pattern was previously described in other osteochondral compartment models on chip [27,40], and is also consistent with pre-clinical *in vitro* studies with IL-1β stimulated chondrocytes [[41], [42], [43]]. Importantly, for the first time, we were able to use an OoC model to replicate sex dimorphisms in the response to inflammatory triggers previously *reported in vitro* [44,45]. Particularly, we found trends towards increased gene expression of *CCL2*, *IL1B* and *IL6* in female osteochondral units, suggesting an increased sensitivity of female cells to inflammatory stimulus.

To further explore the cellular processes taking place in the osteochondral units, we conducted a multiplex array to quantify the levels of secreted inflammatory mediators in the conditioned media of chondrocyte and osteoblast co-cultures. Robust pro-inflammatory mediator secretion was observed after induction of inflammation in osteochondral units. We revealed that the levels of secreted pro-inflammatory cytokines, particularly IL-6, IL-8 and CCL2, can be used to stratify samples isolated from baseline and inflamed osteochondral units. Elevated levels of these proteins provide a microenvironment conductive of immune cell recruitment and catabolic activity [28], a hallmark of OA that is replicated in our osteochondral unit-on-chip model. Interestingly, significant differences in the levels of these mediators are observed between conditioned medium collected from the bone unit (osteoblasts) outlets when compared to cartilage unit (chondrocytes) outlets. These results are consistent with previous studies showing differential response of chondrocytes and osteoblasts in osteochondral interfaces [27,40]. It is well established that chondrocytes and osteoblasts secrete IL-6 in response to IL-1β stimulation [[46], [47], [48]], which contributes to the exacerbated pro-inflammatory environment characteristic of OA. Nevertheless, it cannot be ruled out that the cell source might directly affect the responsiveness of osteoblasts and chondrocytes in our model. Chondrocytes are isolated from total knee replacement surgeries, which might already be pre-conditioned in a highly inflamed microenvironment prior to cell isolation. Osteoblasts, on the other hand, are isolated from healthy bone and have decreased responsiveness to inflammation when compared to osteoblasts from OA subchondral bone [47]. While no sex-specific differences in cytokine production were observed between samples, these results are consistent with our RNA expression assays that show no sex dimorphism in osteochondral units cultured under perfusion. In the future, to consolidate these findings, the number of female and male donors will be expanded to account for the inherent variability of human primary cells. The expansion of our dataset will allow for validation of lead candidates for sexual dimorphism that we identified in this work and yield insights on whether they are generally applicable or if they are representative of only a particular subset of the population.

ECM deposition and remodeling are known to be modulated during OA [32,49]. Thus, it would be important to ascertain whether protein deposition patterns would show significant sex-specific differences after inflammatory stimulus in our model. Metabolic labeling of nascent proteins has previously been used to show that chondrocytes secrete ECM in their pericellular region, which then intersperses and interacts with the pre-existing matrix [31,50]. Metabolic labeling is therefore a useful tool to study the dynamics of protein deposition, but so far it has only been used with 2D confocal images which could overlook variations in nascent protein distribution across the 3D space. We employed an image analysis workflow to detect and segment nascent protein deposition in 3D and found qualitative and quantitative sex-specific differences in nascent protein volume. By extracting cell bounding box dimensions, we were able to automatically crop and quantify nascent protein deposition at a single cell level. In the future, we foresee the integration of this methodology in an analysis workflow for automatic monitoring of protein deposition in longitudinal, live imaging experiments. Future improvements in image acquisition and analysis include the employment of alternative imaging methods with higher tissue penetration such as two-photon microscopy. This would be important to account for hydrogel thickness that can cause signal loss as the laser penetrates further into the hydrogel, leading to intensity differences between z-stacks. Artificial compensation could be used in post-processing, but these procedures assume that signal intensities are similar across the entire hydrogel.

To the best of our knowledge, this is the first report of sexual dimorphism of human chondrocyte protein deposition *in vitro*. Qualitative evidence of sexual dimorphism in protein deposition has been previously provided in a bovine model of articular cartilage [51], while other studies report sexual dimorphism in glycosaminoglycan deposition by human chondrocytes and fibrochondrocytes [52,53]. We also observed different profiles of chondrocyte protein deposition in response to inflammatory triggers when osteochondral units were cultured in static or perfusion conditions, both in terms of secreted/restricted/negative chondrocyte ratios and in protein deposition volume. It is well established that medium perfusion impacts cell metabolism in organ-on-chip platforms when compared to static conditions [[54], [55], [56]]. In our model, diffusion experiments showed increased diffusion of FITC-Dextran under perfusion. Thus, the reduced medium retention time and increased exchange rate of nutrients and metabolites likely explain the observed differences in protein deposition between static and perfusion culture conditions.

As metabolic labeling only provides an overview of deposition of methionine-containing proteins, it needs to be combined with other imaging techniques to identify the ECM components being deposited. Progressive loss of collagen II production and alteration of collagen I deposition is observed in histological samples of OA patients [57], and thus changes in collagen I/II production could explain the decrease in nascent protein deposition observed in the osteochondral units after exposure to inflammatory triggers. Interestingly, we observed differential collagen I/II expression profiles in female and male chondrocytes. Collagen I expression is maintained in female chondrocytes while the number of collagen II expressing cells markedly decreases, which seems to indicate that differences in collagen II expression are not the main driver of alterations in nascent protein volume in female osteochondral units cultured under perfusion. On the other hand, collagen I production in male chondrocytes increases after exposure to inflammation, but this increase is not sufficient to sustain substantial differences in total nascent protein deposition. This trend may be solidified with an increase of power resulting from the inclusion of additional cell donors, yet this expansion falls beyond the scope of the current research goal.

The stiffness of the surrounding matrix has profound implications for the chondrogenic potential and collagen II/GAG deposition of chondrocytes. Matrix stiffnesses ranging from 20 to 30 kPa were reported to maintain chondrocyte round morphology and potentiate differentiation and matrix production when compared to softer matrices [[58], [59], [60]]. We used nanoindentation to measure the stiffness of GelMA directly on-chip, demonstrating the ability of our model to couple molecular biology assays and mechanical characterization studies. Young's modulus of empty hydrogels measured on-chip ranges from 15 to 20 kPa, where the addition of nanoHA to GelMA increases the stiffness by 10 %. Interestingly, Young's modulus arrays in cell laden hydrogels are particularly heterogeneous in both bone and cartilage compartments when compared to empty gels, pointing towards cell-mediated matrix remodeling. Matrix stiffness was significantly reduced after induction of inflammation, consistent with the observed increased gene expression of *MMP1* and *MMP13* and pointing to augmented catabolic activity in both cell compartments. These findings are aligned with clinical data on ECM degradation in OA where proteolytic enzymes that cleave ECM proteins, such as MMP13 and ADAMTS, exacerbate catabolic turnover and lead to the decay of cartilage integrity [2]. Thus, osteochondral units on-chip were able to replicate catabolic degradation and matrix remodeling after exposure to inflammatory triggers, a key hallmark of OA progression [61,62]. In future studies, taking advantage of the high degree of control over fluidic flow in the osteochondral unit-on-chip, a logical improvement of our model would be to include dynamic perfusion of controlled gradients of sex hormones. Since modulation of sex hormones are associated with OA progression [63,64], ascertaining whether loss of e.g.17β-estradiol impacts matrix deposition and remodeling on-chip would be crucial to further explore sex-specific determinants of OA. Another interesting avenue highly compatible with organ-on-chip platforms is to include key components of the immune system, to further investigate potential immune variations between sexes that may critically contribute to the sex differences in the aetiology of OA [65].

## 5 Conclusion

In this study, we replicated the close interactions between osteoblasts and chondrocytes within a 3D microenvironment, thus in a physiologically relevant manner using organ-on-chip technology. The osteochondral unit on-chip model presented here addresses critical limitations of traditional *in vitro* models by: i) encompassing two contiguous, perfusable yet separately addressable cell compartments, one for bone and another for cartilage constructs; ii) incorporating sex-stratified, human primary chondrocytes and osteoblasts; iii) culturing cells in 3D ECM-like matrices, under dynamic conditions; iv) allowing favorable optical access, compatible not only with classical immunocytochemistry but also with emerging technologies such as metabolic labeling; and v) demonstrating feasibility of direct microscale range measurements of matrix mechanical properties on-chip. This system was successfully used to recapitulate sex-specific differences in cellular responses to inflammatory insults, particularly regarding increased gene expression of pro-inflammatory markers and altered matrix deposition and remodeling.

In summary, we have developed a new seamless model of the osteochondral interface to investigate the cellular interactions between human chondrocytes and osteoblasts taking place in a pro-inflammatory microenvironment characteristic of OA. Furthermore, we demonstrated compatibility of osteochondral units on-chip with metabolic labeling and nanoindentation, which could be advantageous in the investigation of underlying mechanisms of not only OA, but also other diseases characterized by altered ECM deposition.

Collectively, our findings set the basis for additional exploration of the sex-specific mechanisms that impact the onset of OA. Further insights on sex-stratified immune modulation could be gained by including sex-matched cellular players from other tissues such as the synovium. In the future, insights gained from sex-stratified osteochondral models could pave the way for the development of novel targeted therapies for OA.

## CRediT authorship contribution statement

**Francisco Conceição:** Writing – review & editing, Writing – original draft, Visualization, Validation, Methodology, Investigation, Formal analysis, Conceptualization. **João Meneses:** Writing – review & editing, Software, Methodology. **Filipa Lebre:** Writing – review & editing, Methodology, Investigation. **Malin Becker:** Writing – review & editing, Methodology, Investigation. **Nuno Araújo-Gomes:** Writing – review & editing, Methodology. **Rianne Vos:** Writing – review & editing, Methodology, Investigation. **Ana R. Ribeiro:** Writing – review & editing, Supervision, Resources. **Ernesto Alfaro-Moreno:** Writing – review & editing, Supervision, Resources. **Jeroen Leijten:** Writing – review & editing, Supervision. **Liliana Moreira Teixeira:** Writing – review & editing, Writing – original draft, Supervision, Project administration, Funding acquisition.

## Declaration of competing interest

The authors declare that they have no known competing financial interests or personal relationships that could have appeared to influence the work reported in this paper.

## Data Availability

Data will be made available on request.
