# Supplementary material for: Sex-stratified osteochondral organ-on-chip model reveals sex-specific responses to inflammatory stimulation
Source: Mater Today Bio. 2025 Apr 2;32:101728. doi: 10.1016/j.mtbio.2025.101728 (PMC12000750; doi:10.1016/j.mtbio.2025.101728)
Supplement: Multimedia component 1 [file mmc1.docx]

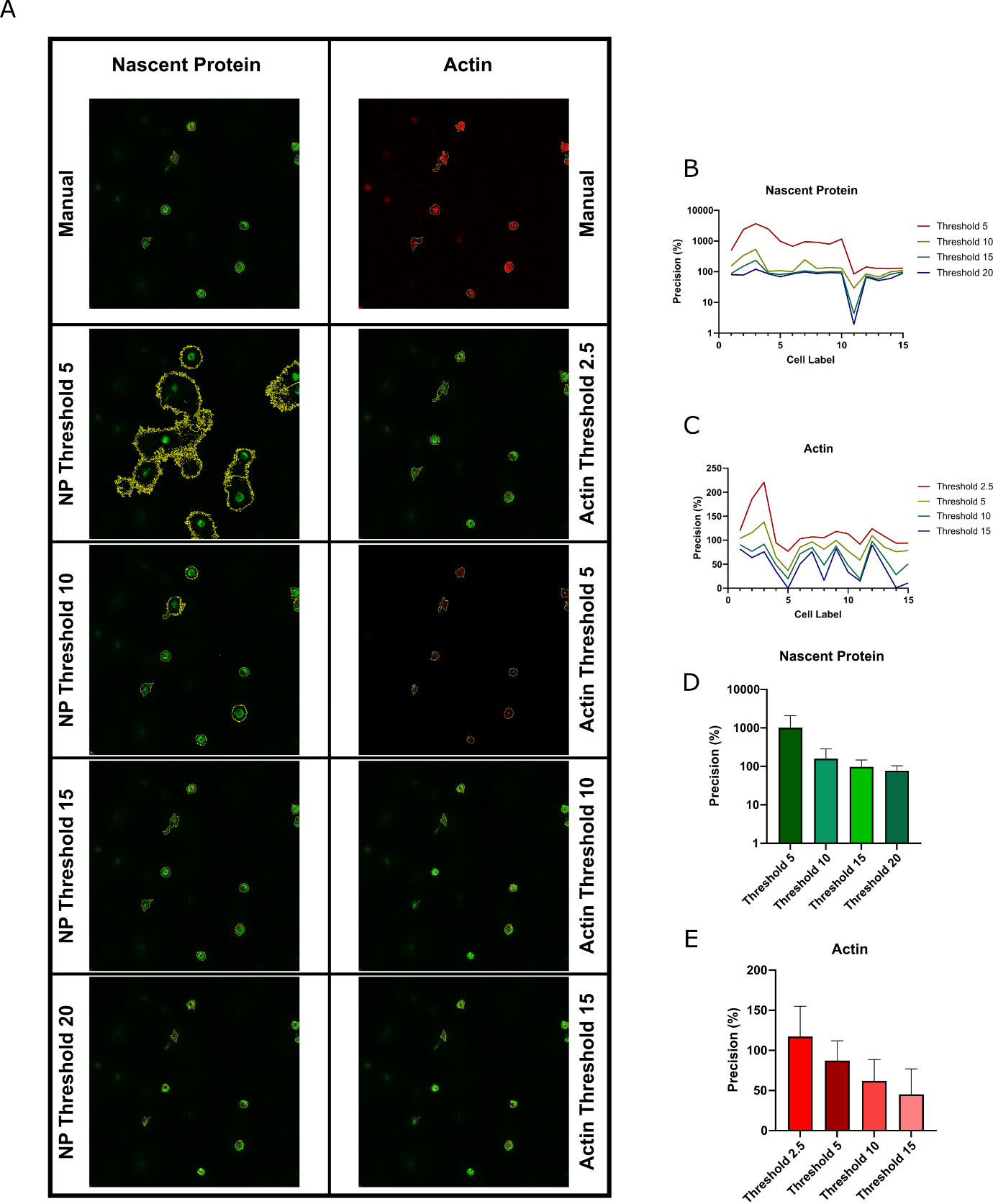
Fig. S1. Thresholding optimization for the automatic quantification of nascent protein and actin volume. A) Representative images of different thresholds and comparison with manual segmentation. B) Graphical representation of the precision of nascent protein segmentation (automatically segmented area divided by manually segmented area multiplied by 100) or C) actin for each detected cell. D) Average nacent protein and E) actin segmentation precision for all different thresholds.


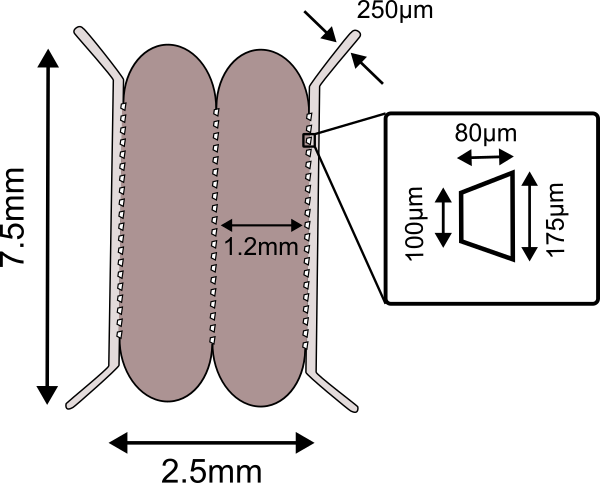


Fig. S2. Osteochondral unit chip device dimensions.


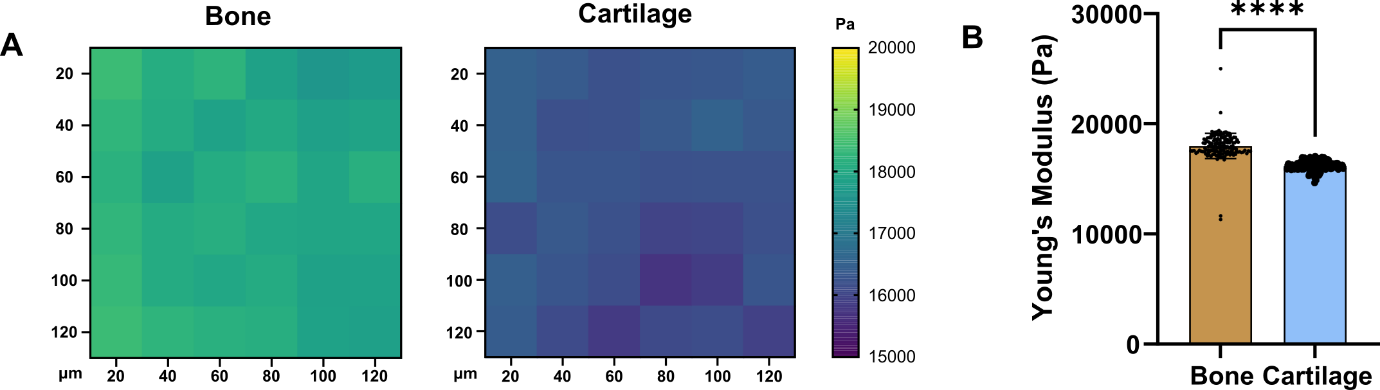


Fig. S3. Mechanical properties of empty hydrogels seeded on chip. A) Representative heatmaps depicting Young’s Modulus array measurements in bone and cartilage compartments. B) Young’s Modulus of empty hydrogels seeded on chip. Data is expressed as mean of individual data points pooled from 4 different arrays (Mann-Whitney test, ****p<0.0001) from one independent experiment.


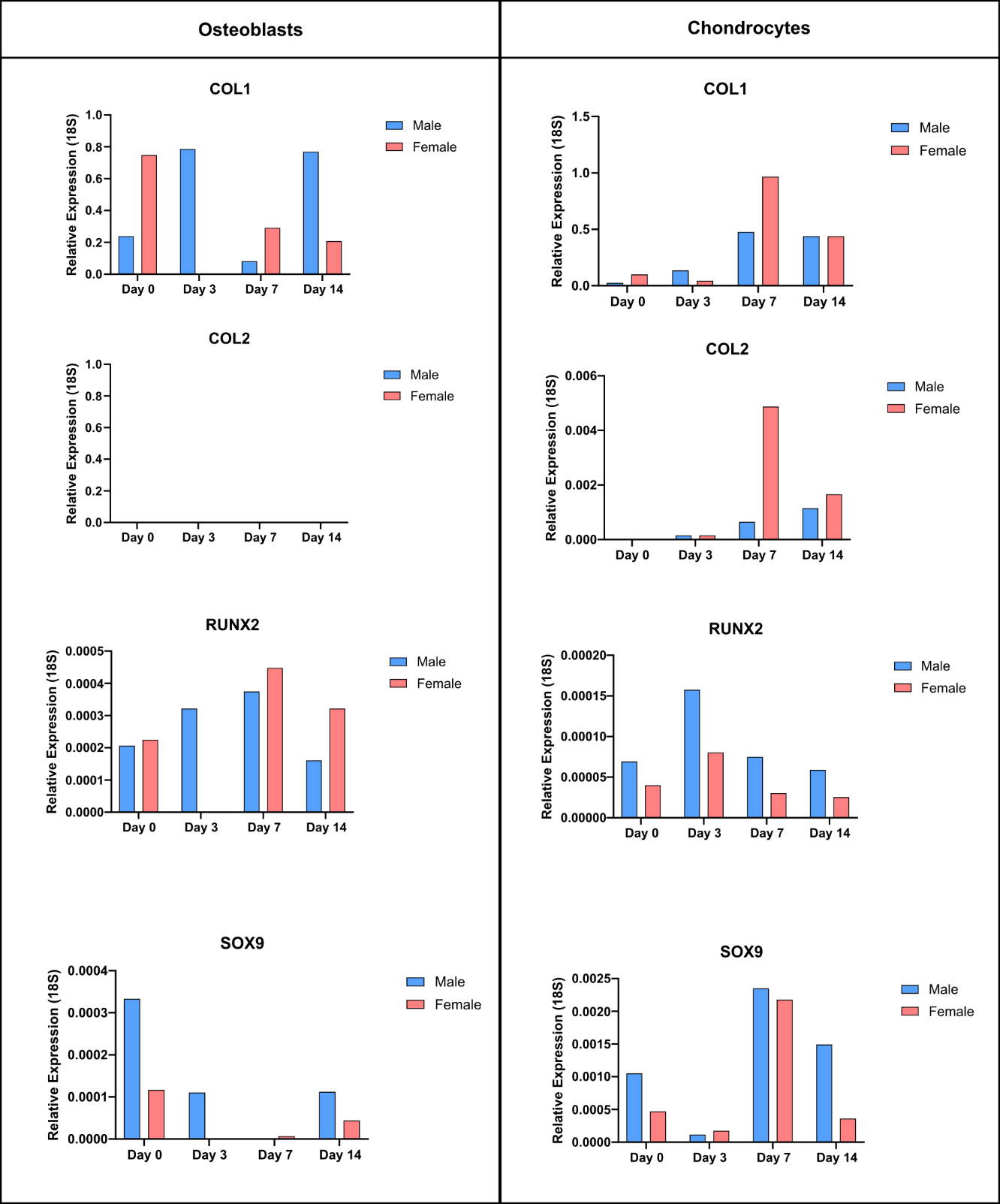


Fig. S4. Gene expression of osteoblast/chondrocyte differentiation markers. Cells were cultured in each respective differentiation medium on 24-well plates for up to 14 days. Data is shown from one independent experiment.


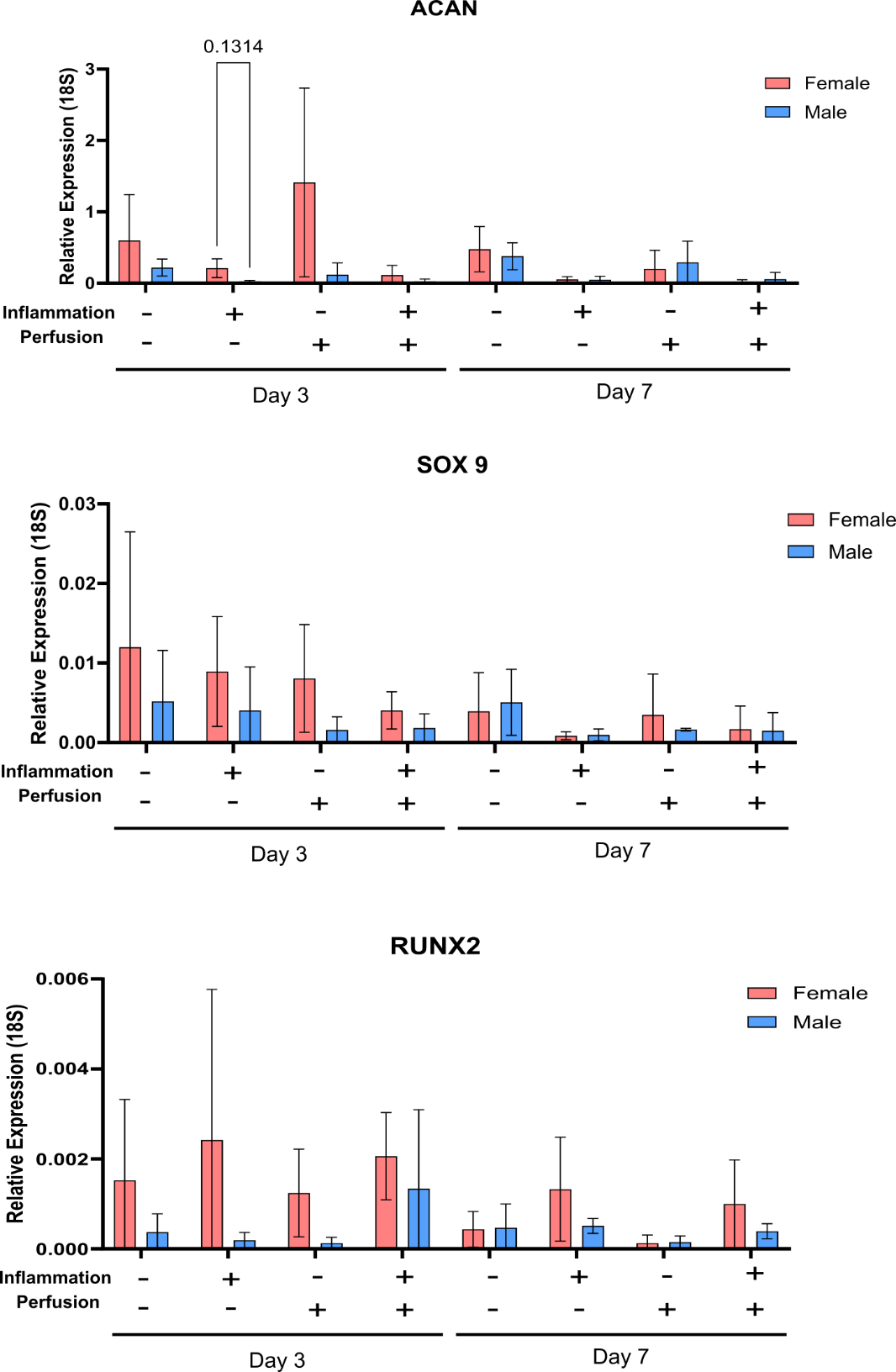


Fig. S5. Differentiation marker gene expression. Gene expression of female and male osteoblast/chondrocyte pairings exposed to control or inflammatory stimulus, cultured either in static or perfusion conditions. Data from three independent experiments was expressed as mean±SD (Two-way ANOVA followed by Tukey’s multiple comparisons test, *p<0.05).


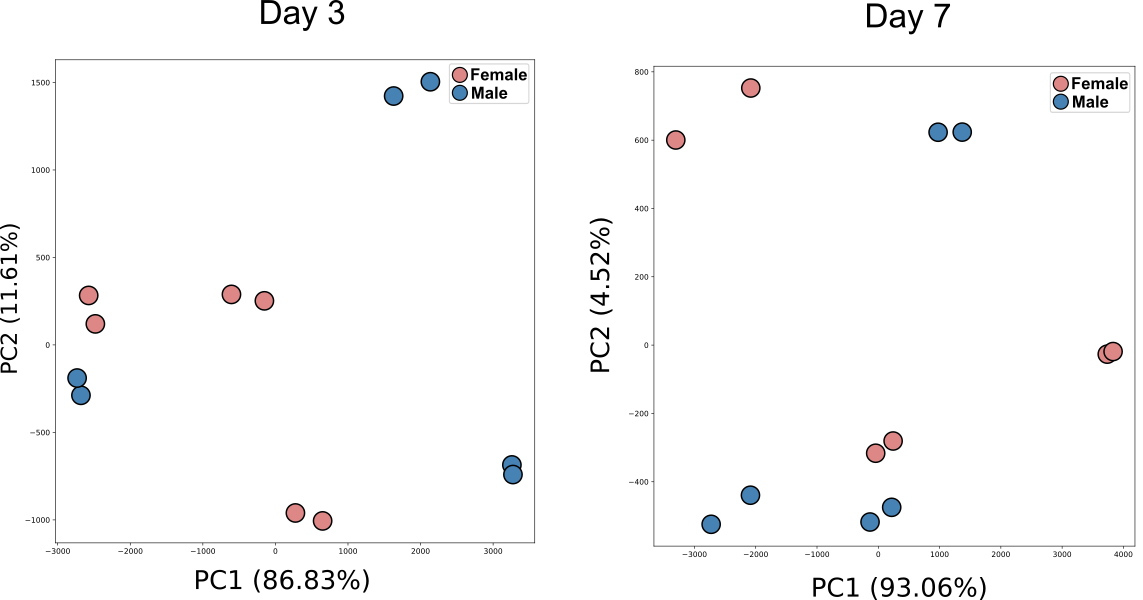


Fig. S6. PCA plot of samples collected from chondrocyte compartments on day 3 (left) and day 7 (right) and stratified by sex.


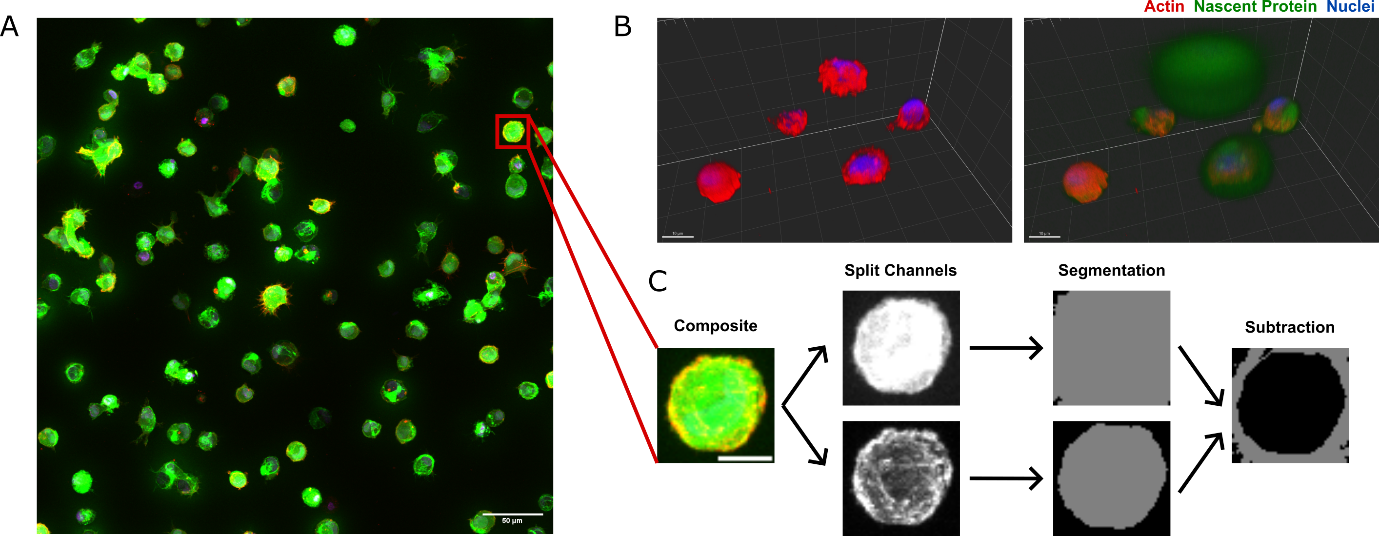


Fig. S7. Schematic depicting nascent protein volume quantification. A) Micrograph of nascent protein staining in chondrocytes cultured for 7 days. Nuclei – Blue; Nascent protein – Green; Actin – Red. Scale bar - 50μm. B) 3D rendering of chondrocytes with nascent protein labeling. Green channel is toggled off in the left image showing clear matrix deposition in the pericellular space. Scale bar - 10μm. C) Rationale for the quantification of secreted matrix in the pericellular space. Composite images are split and segmented independently, after which image subtraction between the two different masks is used to obtain the final mask of pericellular deposition. Images shown are individual stacks for illustration purposes but the analysis is done in 3D. Scale bar 10μm.


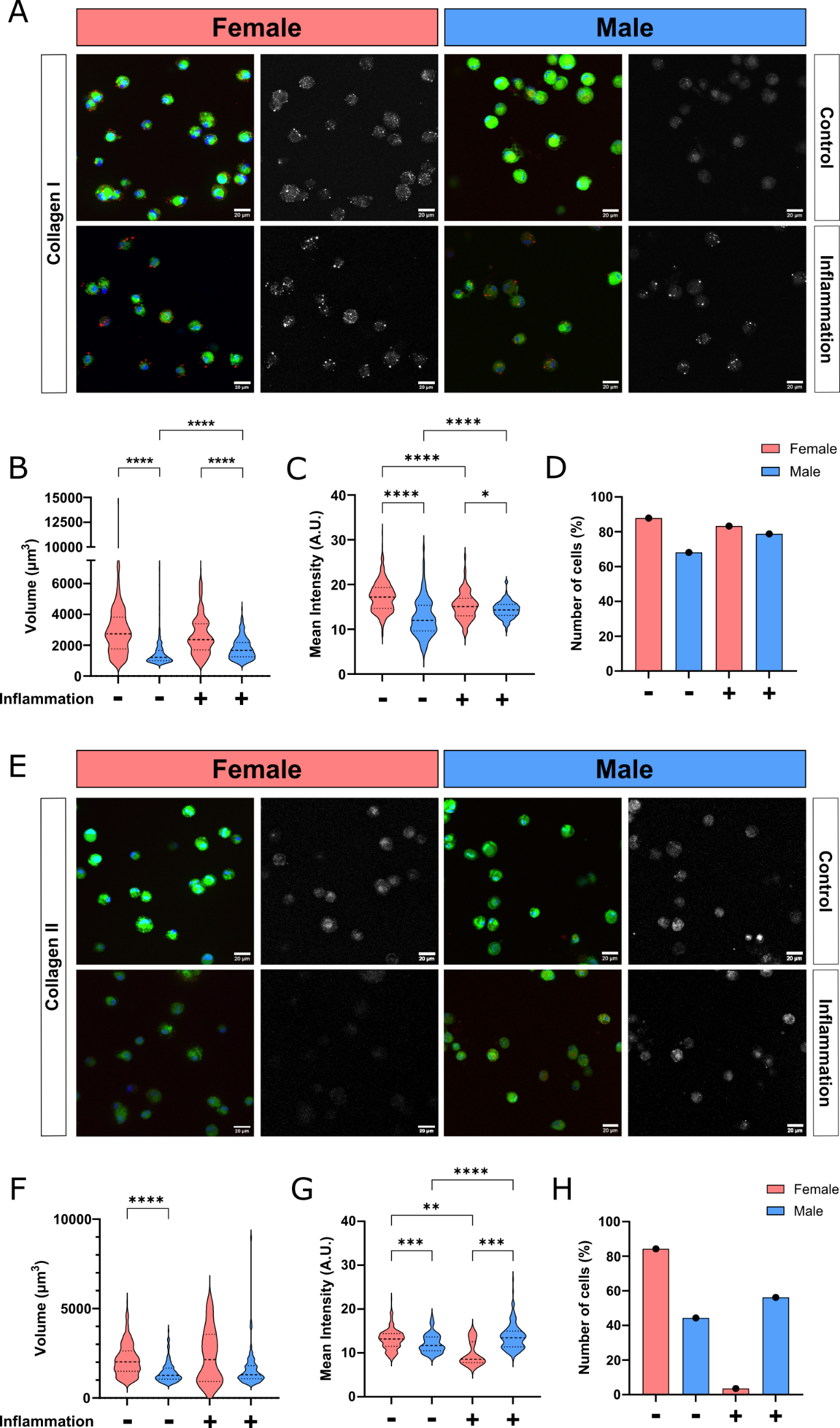


Fig S8. Inflammatory stimulus triggers sex-specific differences in the expression of collagen I and collagen II. A) Representative overview of collagen I expression in female and male chondrocytes, after 7 days of culture under control or inflammatory conditions, under perfusion conditions. Merged images are shown on the left of each panel, grayscale images of collagen I staining are shown on the right of each panel. Nuclei – Blue; Nascent protein – Green; Collagen I - Red. Scale bar - 20μm. B) Total volume of collagen I expressed by female and male chondrocytes cultured under perfusion conditions. Data from more than 250 single cell data points is expressed in violin plots (Kruskal-Wallis followed by Dunn’s multiple comparison test, ****p<0.0001). C) Mean intensity of collagen I signal. Data from more than 250 single cell data points is expressed in violin plots (Kruskal-Wallis followed by Dunn’s multiple comparison test, *p<0.05, **p<0.01, ***p<0.001, ****p<0.0001). D) Quantification of the percentage of cells positive for collagen I, expressed as individual data points. E) Representative overview of collagen II expression in female and male chondrocytes, after 7 days of culture under control or inflammatory conditions, under perfusion conditions. Nuclei – Blue; Nascent protein – Green; Collagen II - Red. Scale bar - 20μm. F) Total volume of collagen II expressed by female and male chondrocytes cultured under perfusion conditions. Data from more than 250 single cell data points is expressed in violin plots (Kruskal-Wallis followed by Dunn’s multiple comparison test, ****p<0.0001). G) Mean intensity of collagen II signal. Data from more than 250 single cell data points is expressed in violin plots (Kruskal-Wallis followed by Dunn’s multiple comparison test, *p<0.05, **p<0.01, ***p<0.001, ****p<0.0001). H) Quantification of the percentage of cells positive for collagen II, expressed as individual data points.

| **Table S1.** List of primers used | | | |
| --- | --- | --- | --- |
| **Gene** | **Accession Number** | **Forward** | **Reverse** |
| ACAN | NM_001135.4 | CACCCCATGCAATTTGAG | AGATCATCACCACACAGTC |
| CCL2 | NM_002982.4 | AGACTAACCCAGAAACATCC | ATTGATTGCATCTGGCTG |
| COL1A1 | NM_000088.4 | GCTATGATGAGAAATCAACCG | TCATCTCCATTCTTTCCAGG |
| COL2A1 | NM_033150.3 | GAAGAGTGGAGACTACTGG | CAGATGTGTTTCTTCTCCTTG |
| CSF1 | NM_000757.6 | TTAAGAAGGCATTTCTCCTG | CCTTGTCATGCTCTTCATAATC |
| ESR1 | NM_000125.4 | GGAGTGTACACATTTCTGTC | CAAAGTGTCTGTGATCTTGTC |
| IGF1 | NM_000618.5 | CCCAGAAGGAAGTACATTTG | GTTTAACAGGTAACTCGTGC |
| IGFR1 | NM_000875.5 | AAAGACAAAATCCCCATCAG | TGCAGGAAATTCTCAAAGAC |
| IL1B | NM_000576.3 | CTAAACAGATGAAGTGCTCC | GGTCATTCTCCTGGAAGG |
| IL6 | NM_000600.5 | GCAGAAAAAAGGCAAAGAATC | CTACATTTGCCGAAGAGC |
| IL8 | NM_000584.4 | GTTTTTGAAGAGGGCTGAG | TTTGCTTGAAGTTTCACTGG |
| MMP1 | NM_001145938.2 | AAAGGGAATAAGTACTGGGC | CAGTGTTTTCCTCAGAAAGAG |
| MMP13 | NM_002427.4 | AGGCTACAACTTGTTTCTTG | AGGTGTAGATAGGAAACATGAG |
| MMP9 | NM_004994.3 | AAGGATGGGAAGTACTGG | GCCCAGAGAAGAAGAAAAG |
| RUNX2 | NM_001015051.4 | AAGCTTGATGACTCTAAACC | TCTGTAATCTGACTCTGTCC |
| SOX9 | NM_000346.4 | CTCTGGAGACTTCTGAACG | AGATGTGCGTCTGCTC |
| SP7 | NM_001173467.3 | TGAGGAGGAAGTTCACTATG | CATTAGTGCTTGTAAAGGGG |
| TGFB1 | NM_000660.7 | AACCCACAACGAAATCTATG | CTTTTAACTTGAGCCTCAGC |
| TGFBR1 | NM_001130916.3 | AGACAATGGTACTTGGACTC | GTACCAACAATCTCCATGTG |
| TNFSF11 | NM_003701.4 | TGGTTCCCATAAAGTGAGTC | GAAGATACTCTGTAGCTAGGTC |
| VEGFA | NM_001204384.2 | AATGTGAATGCAGACCAAAG | GACTTATACCGGGATTTCTTG |
